# Adult schistosomes have an epithelial bacterial population distinct from the surrounding mammalian host blood

**DOI:** 10.1371/journal.pone.0263188

**Published:** 2022-01-27

**Authors:** Geoffrey N. Gobert, Donald P. McManus, Geoff McMullan, Christopher J. Creevey, Jack Carson, Malcolm K. Jones, Sujeevi S. K. Nawaratna, Kosala G. Weerakoon, Hong You

**Affiliations:** 1 School of Biological Sciences, Queen’s University Belfast, Belfast, United Kingdom; 2 Molecular Parasitology Laboratory, Immunology Department, QIMR Berghofer Medical Research Institute, Brisbane, Australia; 3 School of Veterinary Science, University of Queensland, Brisbane, Queensland, Australia; 4 School of Medicine, Griffith University, Gold Coast, Australia; 5 Department of Parasitology, Faculty of Medicine and Allied Sciences, Rajarata University of Sri Lanka, Saliyapura, Sri Lanka; Universidade Guarulhos, BRAZIL

## Abstract

**Background:**

Schistosomiasis is a neglected tropical parasitic and chronic disease affecting hundreds of millions of people. Adult schistosomes reside in the blood stream of the definitive mammalian host. These helminth parasites possess two epithelial surfaces, the tegument and the gastrodermis, both of which interact with the host during immune evasion and in nutrient uptake.

**Methods:**

Female ARC Swiss mice (4–6 weeks old) were infected percutaneously with *Schistosoma japonicum* cercariae freshly shed from *Oncomelania hupensis quadrasi* snails (Philippines strain). Fluorescent *in situ* hybridisation (FISH) was performed by using fresh adult *S*. *japonicum* perfused from those infected mice. Adult *S*. *japonicum* worms were processed to isolate the tegument from the carcass containing the gastrodermis; blood and bile were collected individually from infected and uninfected mice. Total DNA extracted from all those samples were used for microbiome profiling.

**Results:**

FISH and microbiome profiling showed the presence of bacterial populations on two epithelial surfaces of adult worms, suggesting they were distinct not only from the host blood but also from each other. Whereas microbial diversity was reduced overall in the parasite epithelial tissues when compared with that of host blood, specific bacterial taxa, including *Anoxybacillus* and *Escherichia*, were elevated on the tegument. Minimal differences were evident in the microbiome of host blood during an active infection, compared with that of control uninfected blood. However, sampling of bile from infected animals identified some differences compared with controls, including elevated levels of *Limnohabitans*, *Clostridium* and *Curvibacter*.

**Conclusions:**

Using FISH and microbial profiling, we were able to demonstrate, for the first time, that bacteria are presented on the epithelial surfaces of adult schistosomes. These schistosome surface-associated bacteria, which are distinct from the host blood microenvironment, should be considered as a new and important component of the host-schistosome interaction. The importance of individual bacterial species in relation to schistosome parasitism needs further elucidation.

## Introduction

Helminth parasites cause significant chronic disease both in people and in animals of economic importance. Schistosome flukes are blood-dwelling helminth parasites and major pathogens of humans, predominantly in tropical and sub-tropical countries. Currently ~200 million individuals worldwide are afflicted with schistosomiasis [1]. Schistosomes have evolved to utilise host nutritional sources while at the same time avoiding host immunological responses; these activities are two major cornerstones of parasitism. The schistosomes are dioecious and cause significant hepatic immunopathology to their mammalian hosts as a result of fibrotic granulomata produced as the host response to entrapped parasite eggs laid by the paired adult worms [2]. The release of immunomodulatory molecules is one of the strategies employed by schistosomes to aid in their continued parasitism of the mammalian host. Indeed, reports of helminth parasite–host interactions, including GIT (gastro-intestinal trematodes), and their impact on the mammalian host gut microbiome are increasing [3–5]. The human gut microbial changes to *S*. *japonicum* have been recently reported [6], associated infection with the higher abundance of certain taxa. Similarly studies using experimental animals infected with *S*. *japonicum* have also reported gut dysbiosis [7, 8].

For adult schistosomes resident in host blood, immune evasion and the uptake of nutrients are critical for parasite survival. Both functions arise primarily via specialised tissues—the syncytial apical surface tegument and the blind gut lined with an active gastrodermis [9–11]. Characterising the host-parasite interface is an important research avenue for understanding the fundamental biology of these pathogens, and in the identification of new intervention targets. The tegument is one of the cellular targets for the only currently available anti-schistosome drug, praziquantel; and it is also represents a key site for current vaccine design [12]. Both the tegument and gastrodermis have long been the focus of researchers, due to their critical importance in schistosome functional biology [13–16], but new facets are yet to be revealed. For example, how do helminths interact with organisms co-inhabiting the host microenvironment? Which in the case of adult schistosomes, is the mammalian blood stream.

While not directly interacting with adult GIT and blood fluke parasites, or their eggs, the gallbladder is another host organ where the microbiome can be an indicator of host health and physiological discord [17]. The human liver fluke *Opisthorchis viverrini* has been reported to impact on gallbladder health [18], causing biliary lithiasis and potentially carcinogenic outcomes. Although cholecystitis arising from schistosomiasis is a rare complication [19], it does represent a case of concomitant helminthiasis and gallstone disease, presenting as intense inflammation in the gallbladder of infected patients. There is a close association between the gut and the liver of mammalian hosts, both of which are directly damaged by an active schistosome infection [20, 21]. These observations suggest that changes in the contents of the gallbladder may arise during hepatic schistosomiasis.

Bacterial populations are considered ubiquitous in almost all biological settings. The presence or absence of a gastrointestinal helminth infection impacts on the host immune system, leading to an altered host gut microbiome [5, 22]. This has been shown in experimental animal systems, as well as in correlative clinical and interventional studies in endemic countries [22]. The free living nematode *Caenorhabditis elegans* has a diverse bacterial population within the gut of the worm itself, different to the external environment [23]. However, little is known about the occurrence, structure, and function of microbial populations resident within the tissues and organs of parasitic helminths [4, 22]. The direct interaction between adult schistosomes and bacteria has not been explored to a great extent but the recent considerable advances in complementary ‘omics’ technologies, provide a systems approach to investigate schistosome epithelia. These methods can specifically identify the presence and potential importance of bacterial populations associated with blood fluke parasites.

Investigation of modified microbiomes on and/or within helminth parasites is an embryonic area of research and has only been exploited for study of the medically important schistosomes in limited studies. A call to expand the understanding of microbiomes within and on the surface of parasites has been made [4]. Our study is thus the first to consider this novel aspect of biological interplay between schistosomes and bacteria of the host blood microenvironment.

## Methods

### Ethics statement

All work was conducted in accordance with protocols approved by the QIMR Berghofer Medical Research Institute Animal Ethics Committee (P288).

### Parasite isolation and tegument removal

*Oncomelania hupensis quadrasi* snails, harbouring a Philippines (Sorsogon, Luzon) strain of *Schistosoma japonicum*, were maintained at QIMR Berghofer Medical Research Institute. Female ARC Swiss mice (outbred 4–6 weeks old) were infected percutaneously with 60–70 cercariae freshly shed from snails. All the mice were maintained with a clean condition in a negative pressure animal house. All the food, water, nesting material and cages used for mice were sterilized and changed weekly. Mice were monitored by trained staff on a weekly basis for signs of distress, and if present were euthanised.

All mice were used for sample collection. Adult worms (mixed sex) were perfused under sterile conditions at 7 weeks after challenge, with warm (37°C) RPMI 1640 (Invitrogen, Melbourne, Australia) containing no antibiotics. All buffers and equipment used for the perfusion were sterilized and the skin of mice was cleared with 75% ethanol before perfusion process. After perfusion, the schistosome worms were placed into ice cold sterile PBS (purchased sterile freshly opened), counted in a cell culture hood, retained as pairs, and stored in liquid nitrogen. Tegument removal of freshly perfused adult parasites utilised application of the freeze-thaw-vortex method [24] under sterile conditions, including all the buffers, tubes and tips were sterilized and the process performed in a culture hood.

Each tegument or carcasses sample was isolated from the pooling of 120–150 pairs of adult parasites, perfused from 10 infected mice. Fresh worms were frozen in liquid nitrogen followed by thawing on ice and then quickly washing in 400μl of ice-cold TBS (10 mM Tris/HCl, 0.84% NaCl, pH 7.4). The supernatant was removed, a fresh 400μl aliquot of Tris-HCl (10mM, pH 7.4) was added and the tube left on ice for 5 minutes before a final vortex for 5 times with 1 second bursts. The tegument was separated from the carcasses by centrifugation at 1000 x g for 30 min, with the former contained in the supernatant and the latter in the pellet. The tegument-rich supernatant was transferred to another tube and centrifuged for 30 min at 12,000 x g, 4 °C; the supernatant was then discarded, and the tegument-rich pellet re-suspended in 100 μl TrisHCl (10 mM, pH 7.4). The carcass pellet was suspended in 100 μl TrisHCl and lightly homogenized on ice. Total 8 tegument samples and 8 carcasses samples were obtained. Both tegument and carcasses in 100 μl TrisHCl (10 mM pH 7.4) were used for total gDNA (schistosome and bacteria) extraction. This isolation procedure for tegument and carcasses was repeated to obtain distinct biological replicates.

### Collection of host fluids

ARC Swiss mouse hosts were scarified by CO_2_ and the skin was shaved and cleaned in 75% ethanol before cardiac puncture was performed on each mouse under sterile conditions. Approximately 500 μl of whole blood was obtained from each animal; no pooling of blood was undertaken. Blood samples were collected individually from 10 mice infected with *S*. *japonicum* and 10 uninfected mice (as control). The whole gallbladder from each mouse was isolated under sterile conditions and washed 3 times in ice cold sterile PBS. Gallbladder samples were collected individually from 10 mice infected with *S*. *japonicum* and 10 uninfected mice (as control). All samples were stored at -80 °C before gDNA extraction.

### DNA isolation and microbiome profiling

Biological replicate repeats included the collection of blood from 10 infected and 10 uninfected animals; bile from 8 infected and 10 uninfected animals; and tissue from pooled parasites for 6 tegument and 5 carcass preparations. Total DNA of all those samples were extracted by using DNeasy Blood & Tissue Kits (QIAGEN, Hilden, Germany) following the manufacturer’s standard instructions. Mock extraction (no tissue) was used as a kitome control. DNA quality was checked using a NanoDrop spectrophotometer (Thermo Fisher Scientific, Waltham, USA). For 16S rRNA amplicon sequencing of each sample 200 ng DNA was provided, with A260/280 ≥1.8 and A260/230 ≥2.0. For tissues this involved dilutions to 20 μl total volume. The enriched kitome control included a mock isolation of DNA, and the submission of an undiluted 20 μl aliquot.

Microbiomes were generated by 16S rRNA amplicon sequencing (*rrs*) at the AGRF (Australian Genome Research Facility, Brisbane, Australia) using standard protocols. Concentration of samples were confirmed by PicoGreen fluorometry (Invitrogen, Melbourne, Australia) by AGRF before use. The *rrs* V3 and V4 hypervariable regions were amplified using primers 341F and 806R (10mM); Forward sequence: 5’-CCTAYGGGRBGCASCAG-3’, Reverse sequence: 5’-GGACTACNNGGGTATCTAAT-3’. AmpliTaq gold 360 MasterMix (Life Technologies, Carlsbad, USA) was used for the first PCR assay and cycling conditions were as follows: initialisation at 98°C for 30 seconds followed by 30 cycles of 94°C for 10 seconds; 60°C for 10 seconds and 72°C for 30 seconds; followed by a final extension of 72°C for 5 minutes. The PCR reaction volume was 25μl. Thermocycling was completed with an Applied Biosystem 384 Veriti and using Platinum SuperFi mastermix (Life Technologies, Mulgrave, Australia) for the primary PCR. The first stage PCR was cleaned using magnetic beads, and samples were visualised on 2% Sybr Egel (Thermo-Fisher). A secondary PCR to index the amplicons was performed with PrimeSTAR Max (Takara, Japan). The resulting amplicons were cleaned again using magnetic beads, quantified by fluorometry (Promega Quantifluor) and normalised. The equimolar pool was cleaned a final time using magnetic beads to concentrate the pool and then measured using a High-Sensitivity D1000 Tape on an Agilent 2200 TapeStation. The pool was diluted to 5nM and molarity was confirmed again using a Qubit High Sensitivity dsDNA assay (ThermoFisher). This was followed by sequencing on an Illumina MiSeq (San Diego, CA, USA) with a V3, 600 cycle kit (2 x 300 base pairs paired-end).

Paired-ends reads were assembled by aligning the forward and reverse reads using PEAR (version 0.9.5) [25]. Primers were identified & trimmed. Trimmed sequences were processed using Quantitative Insights into Microbial Ecology (QIIME 1.8) [26] USEARCH (version 7.1.1090) [27, 28] and UPARSE [29] software. Using USEARCH sequences were quality filtered, full length duplicate sequences were removed and sorted by abundance. Singletons or unique reads in the data set were discarded. Sequences were clustered followed by chimera filtered using “rdp_gold” database as the reference. To obtain the number of reads in each Operational Taxonomic Unit (OTU), reads were mapped back to OTUs with a minimum identity of 97%. Using QIIME taxonomy was assigned using Greengenes database [30] (version 13_8, Aug 2013, 97% sequence similarity cut-off) and rarefied to a sequence depth of 28,700. Rare OTUs with <5 assigned amplicon sequences or <0.0001 fraction of total sequence reads were excluded and OTU reference sequences were aligned using pynast. The trimmed multiple alignments were then used to infer a phylogenetic tree using fasttree, which was then used as the input for UniFrac to estimate microbial beta diversity.

### Data analysis

The presence of the negative kitome control allowed the use of the decontam package [31] in R to identify OTUs that should be filtered using the ‘prevalence’ method with a threshold of 50%. All OTUs identified as possible contaminants from this analysis were removed using the BIOM toolkit [32]. This filtered dataset was used for all subsequent analyses.

Calypso (version 8.54) [33] was used for mining the *rrs* microbiome dataset and for data visualisation.

Rarefied and filtered OTU tables were uploaded to Calypso and square root (SQR) transformed with TSS (total sum normalisation). Samples with less than 1,500 reads were removed. Microbial species diversity was characterised using the Simpson, Chao1, Shannon and Richness diversity indices. Correlations with continuous variables (such as tissue type or tissue and infection status) were calculated using ANOVA. Significant taxa were identified by ANOVA. Fold Change classifications for taxa follows with “#NAME?” = Sample A has no abundance detected; “Inf” = Sample B has no abundance detected; and “NaN” = Both Samples have no abundance detected. The later classification for a taxa is include despite no abundance in the samples selected, due to its presence (abundance) in other samples. With statistical significance considered at *p-value*≤0.05, while not significant is indicated by *NS*.

Multivariate data visualization and multivariate statistical testing were used to indicate the presence of multifaceted associations between microbial communities. Both supervised Redundancy Analysis (RDA) and Canonical Correspondence Analysis (CCA) and unsupervised Principal Coordinates Analysis (PCoA) methods were used at the OUT level unless noted. Both Calypso and biome files before and after normalisation of the kitome are available (see below).

### Fluorescent *in situ* hybridisation (FISH)

Adult *S*. *japonicum* were fixed in 4% (w/v) paraformaldehyde for 24 hours and then embedded in paraffin blocks. Sections (4 μm) were prepared as described [34] and probed with a universal bacterial label. This consisted of the 16*S* rRNA gene CalFluor double-labelled probe Eubacteria EUB338 (5’Calfluor590-GCTGCCTCCCGTAGGAGT-3’, Sigma-Aldrich, St. Louis, USA), and was specific for the domain *Bacteria* [34]. A nonsense probe, Non-EUB338 [35], which has a nucleotide sequence complementary to the nucleotide sequence of EUB338 and was synthesised by IDT (Integrated DNA Technologies), was used as a control for nonspecific staining. The use of no probe was also performed as a negative control. Hybridization was performed by incubating rehydrated samples with PBTA-HB 1:1 (PBTA: phosphate buffered saline with 0.05% BSA and 0.2% Triton X-100, 0.02% sodium azide; HB:hybridisation buffer: Tris-HCl 0.02 M, Sodium Chloride 0.09 M, SDS 0.01%, Formamide 35%, Denhardt’s solution 15%) for 20 min followed by only hybridisation buffer (500 μl/tube), sonication in an ultrasonic bath for 20 s, and the addition of probes and corresponding helpers (final 100 pmol each). A no probes control was also included. Samples were incubated at 45°C in total darkness for 20 h, then washed for 1 h with hybridisation buffer at room temperature, followed by a brief wash in PBTA. Samples were mounted with PBS:glycerol mounting medium. The tissue sections were visualized under fluorescence using a Zeiss780NLO confocal microscope (Zeiss, Oberkochen, Germany).

### Availability of data and materials

The annotation metadata file and bacterial profiles in Calypso v3 format csv (comma-separated values) and biome files (before and after kitome normalisation), are available as (S1 Table and S1–S4 Files). S3 and S4 Files *.biome cannot be opened by standard Microsoft software. S1 and S2 Files represent a conversion of these biome files by Calypso into a format that can be easily reviewed in Excel.

## Results

### *In situ* analysis of the parasites

**Fig 1:**
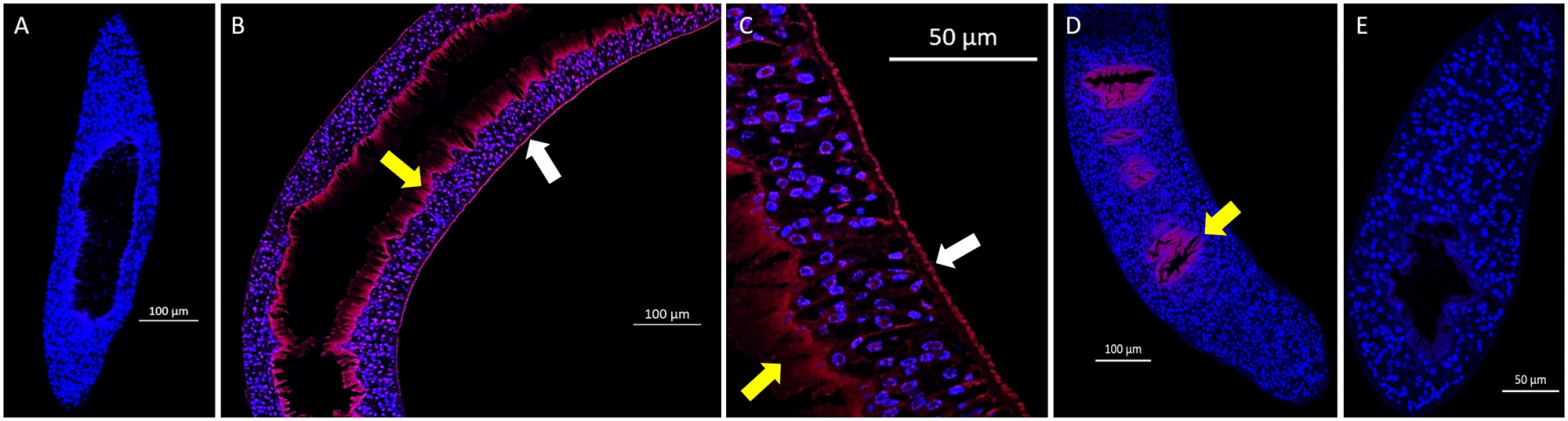
Generic Fluorescent *in situ* hybridisation (FISH) of adult *S*. *japonicum* for bacteria. **A.** No probe negative control. **B.** and **C.** Positive (red) labelling of the female parasite tegument (white arrows) and gastrodermis (yellow arrows) using probe Eubacteria EUB338 (Calfluor590). **D.** Positive (red) labelling of the male parasite gastrodermis (yellow arrows) using EUB338 probe. **E.** Non-EUB338 negative control. Cell nuclei are stained blue by DAPI.

A mouse model of *S*. *japonicum* was used to compare microbial populations within host blood and bile during an active parasite infection, and those of adult worms isolated from host blood. To demonstrate that the two parasite epithelial surfaces, the tegument and gastrodermis, both harboured bacterial populations, we used a generic FISH probe, EUB338, specific for the Superkingdom *Bacteria*, using sections from whole intact adult worms (Fig 1). Strong labelling was noted both on the tegument (white arrow) and gastrodermis (yellow arrow) of adult female *S*. *japonicum*, whereas the EUB338 signal was only observed on the gastrodermis (yellow arrow) of male parasites. Lighter labelling was evident within the parasite tissues potentially indicating the presence of bacteria or that the probe may have bound non-specifically, but to a much lesser degree, to the parasite.

### Sample size and overall microbiome comparison of all tissues

For the molecular analysis, adult parasites were perfused from mice under sterile conditions, washed and the tegument (apical epithelial surface) removed by a non-detergent freeze/thaw/vortex method, used routinely by our and other groups [36, 37], with the remaining carcass separated from the tegument by centrifugation. Thus, two sub-regions of the adult parasite could be examined separately: (i) the surface tegument and (ii) the carcass containing the gastrodermis. Both the tegument and gastrodermis are in direct contact with host blood in which the adult parasites reside.

We performed bacterial profiling using deep amplicon sequencing of the bacterial 16S rRNA gene. Host samples that were retained with sufficient reads included blood from 10 infected mice (M blood-I), blood from 10 uninfected mice (M blood-U), bile from 8 infected mice (M bile-I) and bile from 10 uninfected mice (M bile-U). Parasite samples retained for analysis included tegument from 6 pooled parasite preparations (Sj-Tegument) and carcasses from 5 pooled parasite preparations (Sj-Carcass). Samples were not analysed further if gDNA isolations did not provide material of appropriate standard. An enriched kitome negative control from a mock DNA isolation was also included and a similar volume of material was used for 16S rRNA profiling. The impact of subtracting the prominent OTUs (Operational taxonomic unit) present in the kitome from all other samples, is presented in S1 Fig. The resulting removal of major kitome OTUs allows for the more effective identification of taxa associated with individual tissues. Subsequently, the online bioinformatics analysis tool Calypso [33] was used to characterise the microbial communities across the different tissue sources examined.

**Fig 2:**
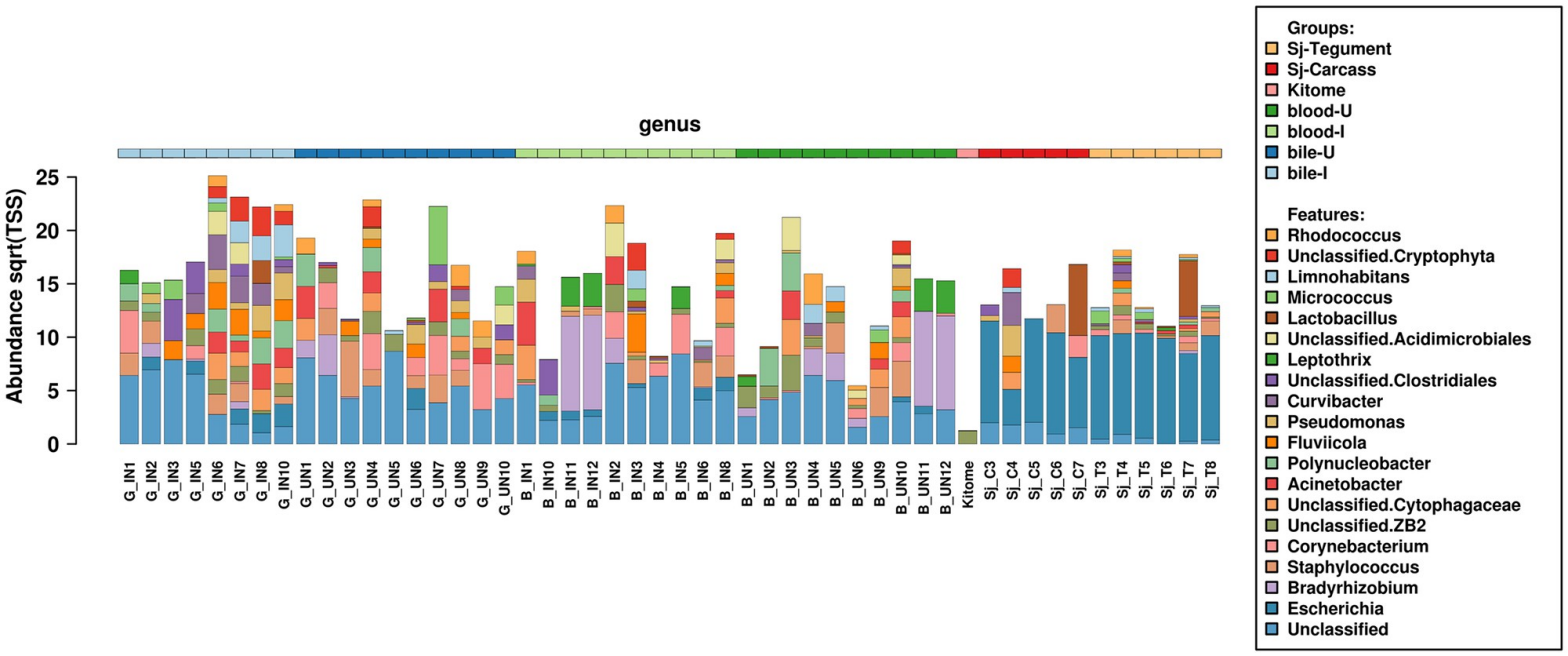
Major bacterial genera associated with parasite tissues or host fluids presented as a non-clustered bar-chart. This graph shows a clear tissue demarcation, particularly between host (left) and parasite (right). The y-axis presents the abundance of each taxa and the x-axis shows the individual samples profiled. Grouping by tissue source is colour-coded at the top.

Quantitative diversity of tissue and control samples is presented in Fig 2 as a non-clustered bar-chart, with an overview of samples grouped by tissue and infection types. Separation between host and parasite tissues continues up to, but not including, the phylum level of microbe profiling (S2 Fig).

**Fig 3:**
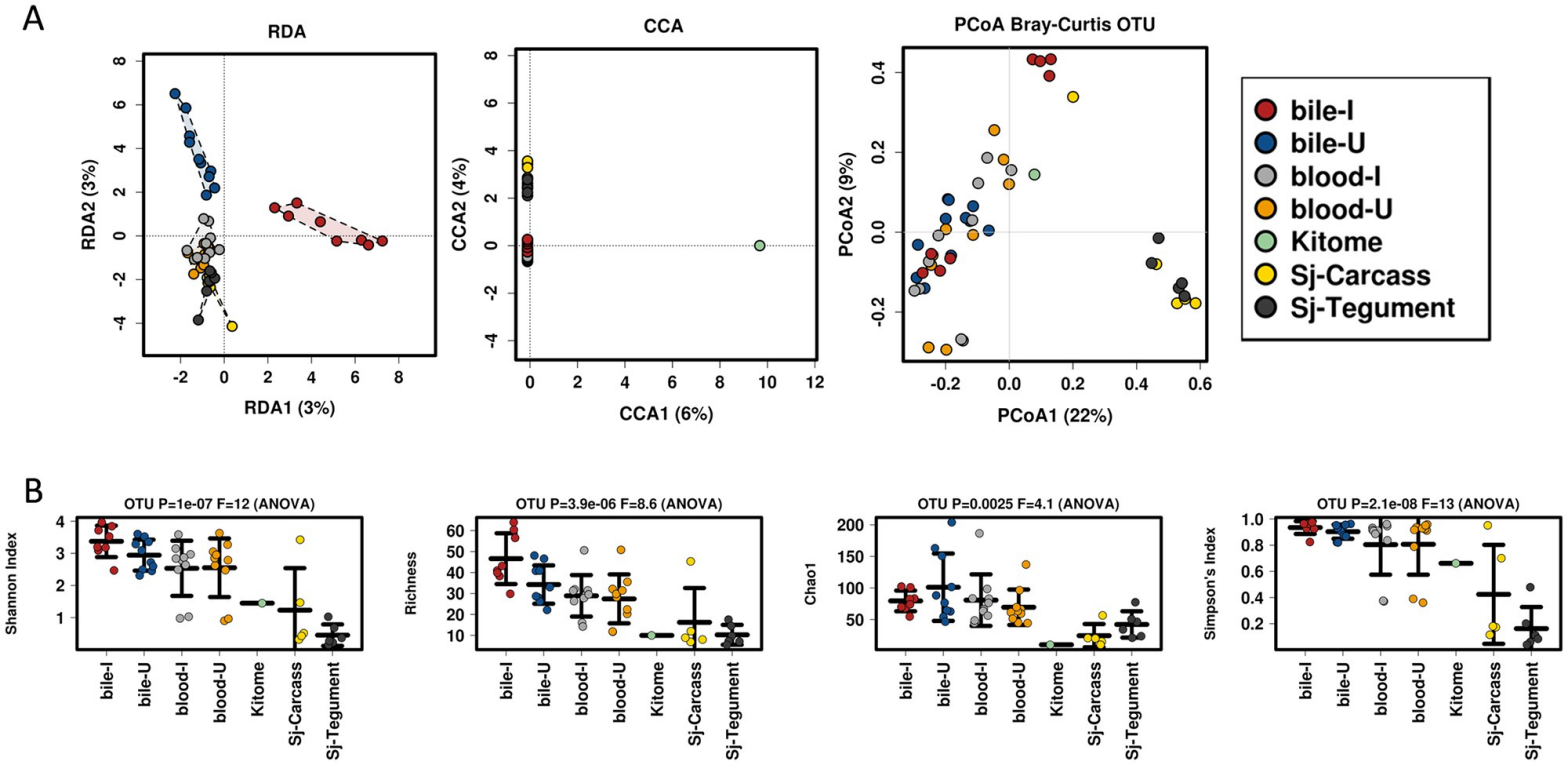
Analysis of all control, mouse and parasite samples, using OTUs. **A.** Multivariate analysis using RDA, CCA and PCoA, respectively. CCA demonstrated statistical differences (*p-value* = 0.001), between the kitome and other samples. **B.** All four diversity analysis methods indicated statistical differences between the samples.

The relationships between host and parasite samples are demonstrated by RDA, CCA and PCoA (Bray-Curtis) Multivariate Analyses and are presented in Fig 3. We noted the kitome was distinct from the other samples in all comparisons (CCA *p-value* = 0.001). Samples from mouse blood and bile, as well as the parasite sources were profiled further for microbiome comparisons. Multivariate data visualization and multivariate statistical testing were used to show multifaceted associations between microbial communities associated with the tissue sources examined. Multiple approaches for determining microbial diversity (Fig 3B) were employed to compare the tissues examined. Shannon index, richness, Simpson index and Chao 1 were used, and all indicated statistically significant differences between the host and parasite tissues.

### Comparison of microbiomes associated with host blood and bile

**Fig 4:**
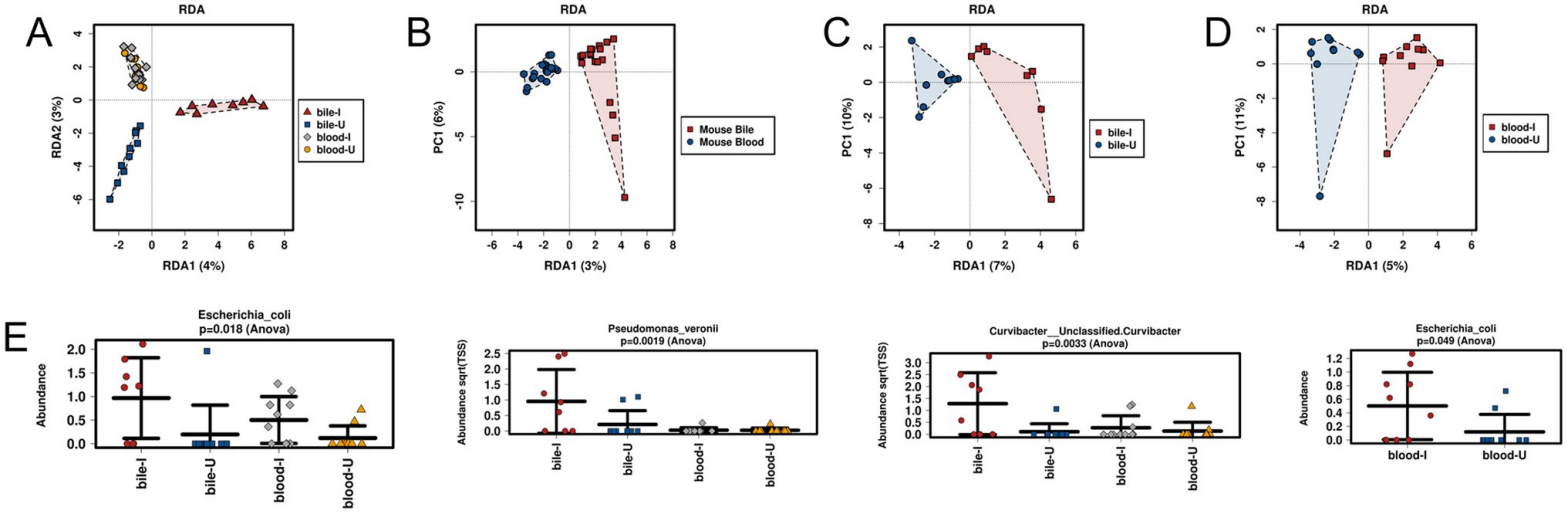
Analysis of blood and bile samples. RDA Multivariate Analysis of **A.** blood and bile by infection status, **B.** blood and bile samples collectively (*p-value* = 0.022), **C.** bile only by infection status (*p-value* = 0.033) and **D.** blood only by infection status. A statistical difference was noted only between blood and bile samples when considered collectively without infection status, and for bile from infected and uninfected hosts. **E.** Examples of species with differential abundance between blood and bile samples. The complete list of species with ANOVA is presented in S2 Table.

The host blood and bile samples were subjected to additional analysis to further characterise the impact of infection on the microbiome of these fluids. RDA (Fig 4A) and CCA did not indicate any statistical difference between the two fluids including the presence or absence of *S*. *japonicum* infection. However, additional RDA comparisons of blood and bile collectively (Fig 4B *p-value* = 0.022), and infection status of bile only (Fig 4C *p-value* = 0.033), were both statistically significantly diverged, although the blood microbiome based on infection status was not (Fig 4D, *NS*).

ANOVA indicated many taxa differed between bile and blood (Fig 4E), which is unsurprising due to the differences in these fluid types. Complete lists of species, genera and phyla tested by ANOVA are presented in S2 Table. The most biologically relevant comparison in terms of the impact parasites have on the host microbiome was performed on specific fluids and their infection status. In the analysis of blood only based on infection status, a single species (*Escherichia coli*) was elevated in blood from infected mice (Fig 4E, *p-value* = 0.049) compared with blood from uninfected animals.

**Fig 5:**
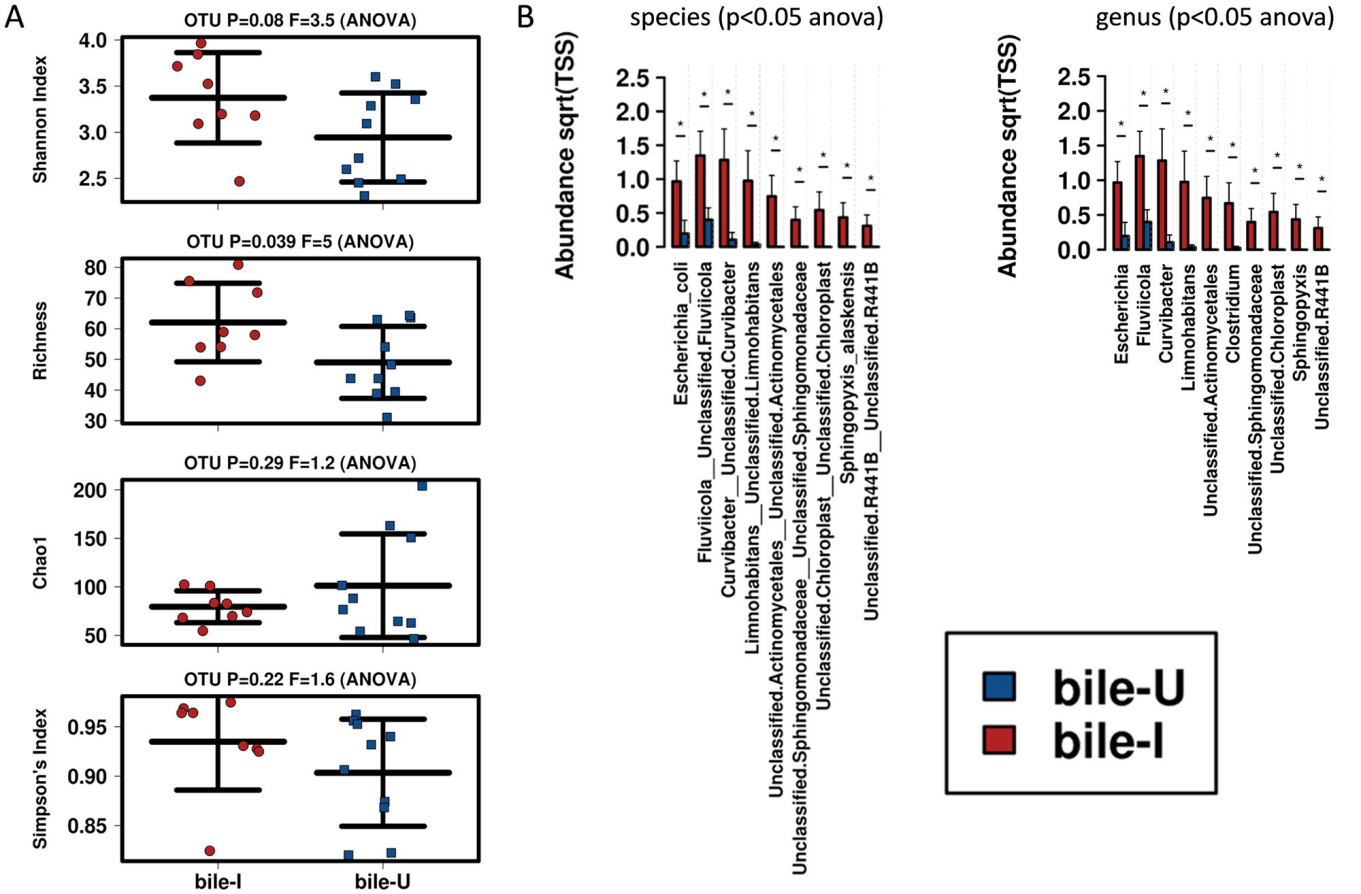
Analysis of bile by infection status. **A.** Diversity showed statistical differences using Richness metric (*p-value =* 0.039) only. **B.** Species and genus were identified by grouped ANOVA with increased levels associated with bile from infected animals. The y-axis presents the abundance and the x-axis shows specific taxa. * = p-value≤0.05. Complete ANOVA lists are available in S3 Table.

In addition to the changes observed in the blood samples with infection status, microbiome diversity between bile from infected and uninfected mice was statistically different in one of the four metrics used (Richness *p-value* = 0.039, Fig 5A). Multiple differences were evident at the species and genus levels (Fig 5B), with a general increase in taxa abundance associated with bile from infected animals. Examples of genera with increased abundant in the bile of infected animals included *Curvibacter* (*p-value* = 0.013, 12 fold); *Fluviicola* (*p-value* = 0.022, 3.4 fold); *Clostridium* (*p-value* = 0.026, 28 fold); and *Limnohabitans p-value* = 0.031, 29 fold). Complete lists analysed by ANOVA are presented in S3 Table. There were limited examples of decreased bacterial species present in bile from infected mice and none were statistically significant.

### Comparison of microbiomes associated with parasite tissues and host blood

**Fig 6:**
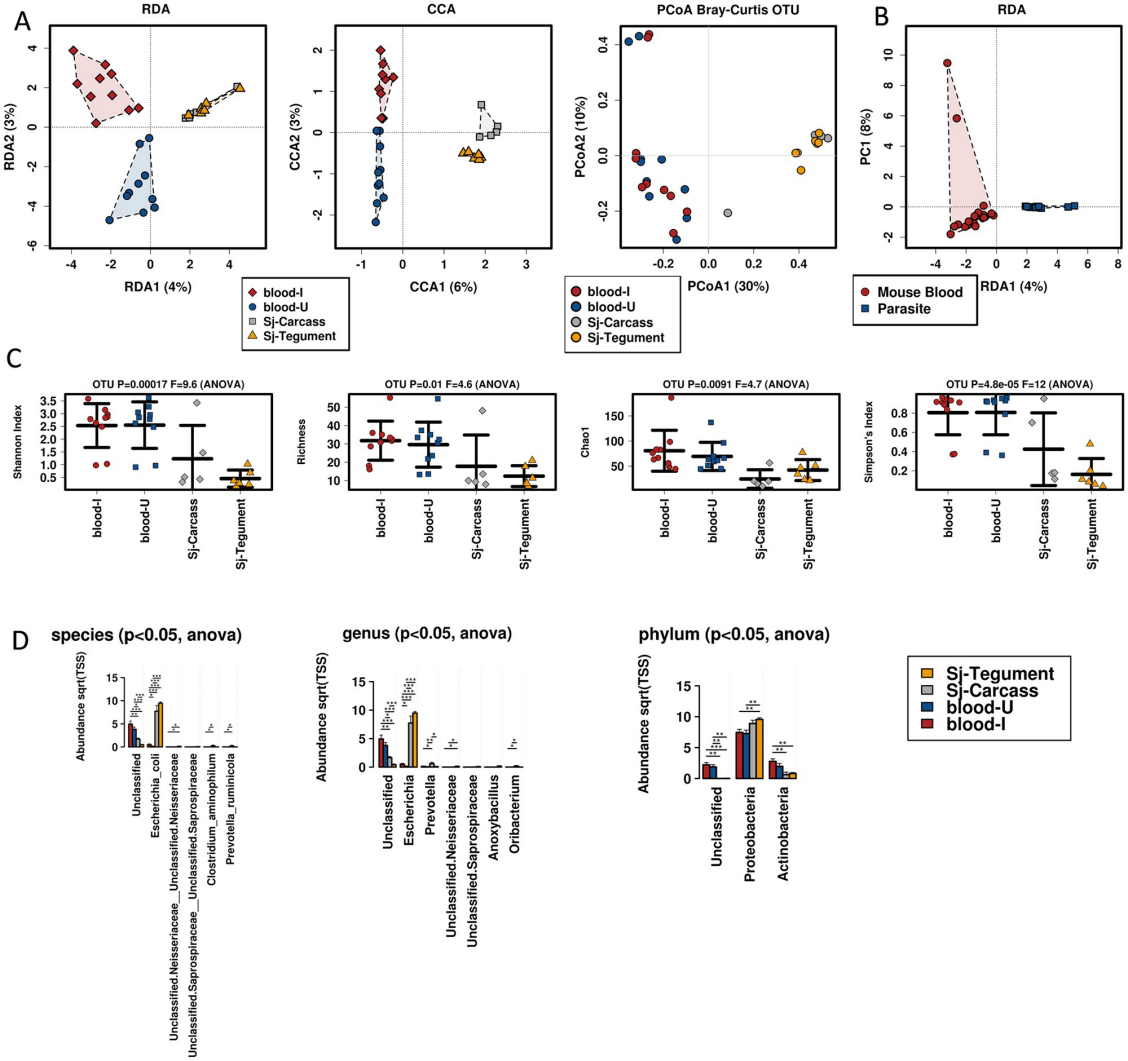
Comparison of the microbiomes of parasite tissues and host blood from infected and uninfected mice. **A.** Multivariate analysis of blood from infected and uninfected hosts, and tegument and carcass tissues from *S*. *japonicum* using RDA and CCA (*p-value* = 0.007) and PCoA (Bray-Curtis) displays overall relationships of the blood samples and parasite tissues. **B.** Comparison by RDA of parasite tissues combined and blood tissue collectively indicates statistically significant groupings (*p-value* = 0.007). **C.** All four diversity metrics indicated statistical differences between the blood samples and parasite tissues, with less diversity evident with the *S*. *japonicum* tissues. **D.** Grouped ANOVA identified species, genera and phyla with increased levels of bacteria associated with both the blood samples and parasite tissues. The y-axis presents the abundance and the x-axis shows specific taxa. * = p-value≤0.05, ** = p-value≤0.01, *** = p-value≤0.001. Complete ANOVA lists are available in S4 Table.

The microbiomes of *S*. *japonicum* tissue sources, and blood from infected and uninfected mice were determined. Differences between these microbiomes were clear as is shown in Fig 6A and 6B. CCA between the four tissue types (*p-value* = 0.007); and the RDA comparison between parasite and host blood both combined (*p-value* = 0.007) were statistically different. These differences were also reflected in diversity metrics (Fig 6C) and specific examples at the species, genus and phylum taxa levels (Fig 6D) were apparent. Examples of taxa elevated either in host blood or parasite tissues are presented. Genera enriched either on the tegument and/or the carcass included *Escherichia* (*p-value =* 4.50E-16); *Unclassified*.*Neisseriaceae* (*p-value =* 0.0034); *Anoxybacillus* (*p-value =* 0.027); *Unclassified*.*Saprospiraceae* (*p-value =* 0.036); *Prevotella* (*p-value =* 0.0054); and *Oribacterium* (*p-value =* 0.0096). Examples of phyla presenting decreased abundance on the parasite epithelial layers when compared to the surrounding blood (infected and uninfected) included Unclassified (*p-value =* 0.000039); and Actinobacteria (*p-value =* 0.0059). Complete ANOVA lists are available in S4 Table. All four diversity metrics indicated statistically robust differences, with a consistent trend that *S*. *japonicum* tissues had less bacterial diversity compared with the host blood in which they reside.

The comparison of parasite tissues and host blood revealed signature microbiomes for one or more tissue/fluid type. S3 Fig presents further examples of species with elevated levels either in the host blood, or both parasite tissues, or specifically in the parasite tegument or carcass. Examples of tegument enriched taxa included *Escherichia_coli* (*p-value* = 4.5E-16), Unclassified.*Neisseriaceae* (*p-value* = 0.0034), and Unclassified.*Saprospiraceae* (*p-value* = 0.036).

### Comparison of microbiomes of the parasite tegument and carcass (gastrodermis)

**Fig 7:**
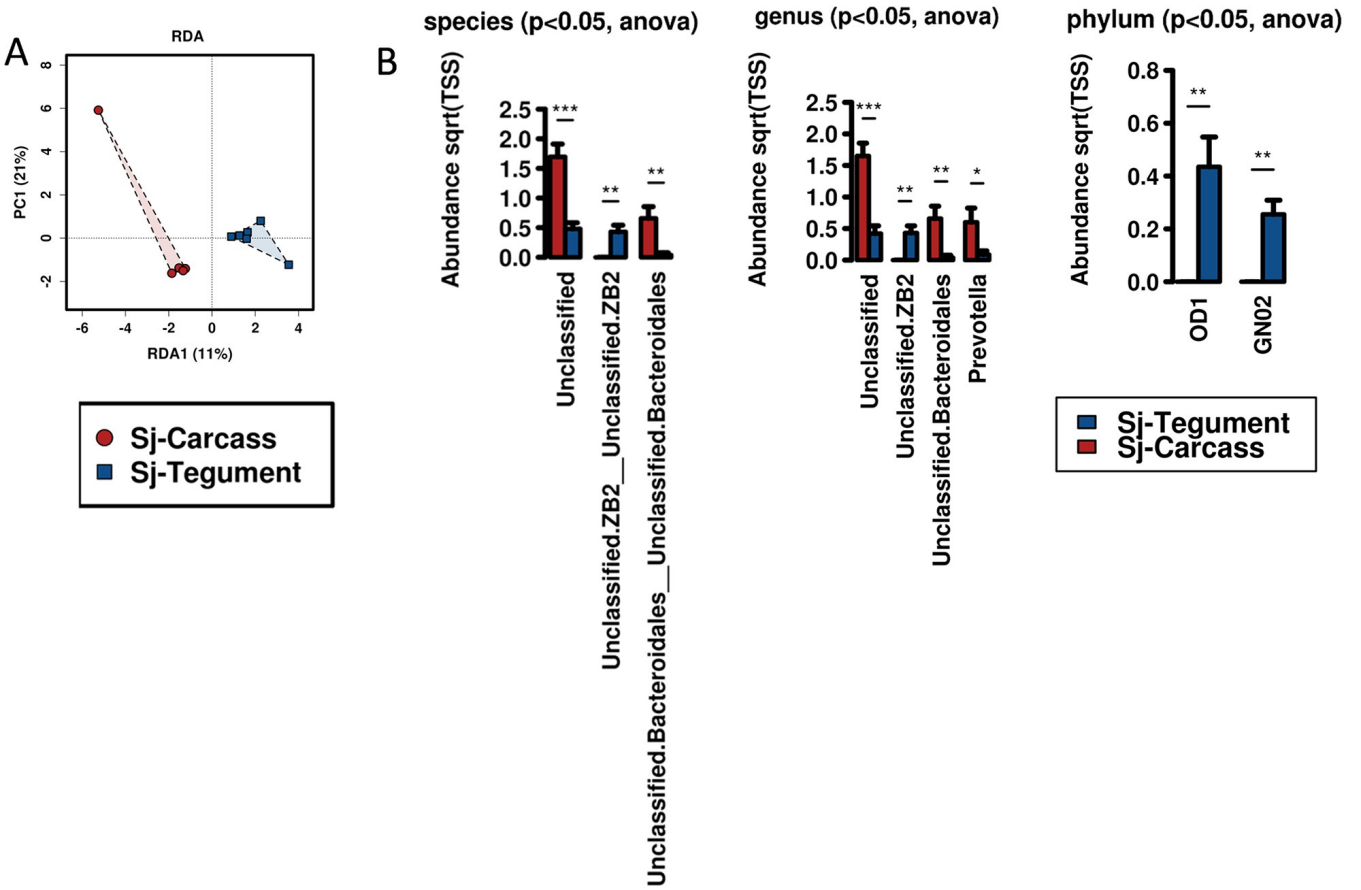
Comparison of the tegument and carcass (gastrodermis) microbiomes of *S*. *japonicum*. **A.** Multivariate analysis of the parasite tissues by RDA demonstrated statistical differences (*p-value* = 0.03). **B**. Grouped ANOVA identified species, genus and phylum with increased levels associated with the parasite tegument compared with the carcass. The y-axis presents the abundance and the x-axis shows specific taxa. * = *p-value*≤0.05, ** = *p-value*≤0.01. Complete ANOVA lists are available in S5 Table.

The tegument and carcass (gastrodermis) microbiomes were considered separately. While the two parasite tissues presented as two groups by multivariate analysis (Fig 7A, RDA, *p-value* = 0.03), bacterial diversity was similar across all four metrics presenting with non-significant differences. Grouped ANOVA (Fig 7B) reflected at the species, genus and phylum levels, examples of elevated taxa associated with both the carcass and the tegument. Complete ANOVA lists are available in S5 Table. Genera observed were generally considered unclassified, increased on the tegument was *Unclassified*.*ZB2 p-value* = 0.0069, while more taxa were elevated in the parasite carcass Unclassified *p-value* = 0.00044; Unclassified. Bacteroidales *p-value* = 0.0078; and Prevotella *p-value* = 0.039.

## Discussion

### Direct interactions of bacteria with helminths

The presence and analysis of bacteria directly associated with blood-dwelling parasites have attracted less attraction compared to that with soil-transmitted helminths. The carcinogenic liver fluke *Opisthorchis viverrini* was cultured after *in vivo* isolation and its microbiome profiled [38]. Aerobic conditions of cultured worms produced bacterial growth predominantly of *Aggregatibacter* and *Lactobacillus*. Liver tissue from normal and *O*. *viverrini-*infected animals also identified bacterial populations including *Acidaminococcus*, *Aggregatibacter*, *Bifidobacterium*, *Clostridium*, *Escherichia*, *Fusobacterium*, *Lactobacillus*, *Megasphaera*, *Streptococcus* and *Veillonella* [38].

Other helminths have been shown to not only alter the host microbiome, but also to potentially modify their own microbiota, based on *in vivo* animal studies of *Trichuris muris* [34]; White and colleagues demonstrated that *T*. *muris* differentiated its internal microbiome from that of the host gut. The infection itself also altered the host gut microbiome by decreasing the abundance of *Bacteroidetes*, while increasing *Firmicutes*, with an overall lowering of bacterial diversity. Examination of the microbiome within the parasite itself identified similar levels of previously reported phyla associated with the host, but surprisingly an increase in levels of *Proteobacteria*, indicating a microbiome distinct to the host. The authors of this *Trichuris* study [34] suggested that the potential presence of higher levels of O_2_ present in the parasite gut may provide a better environment for the *Proteobacteria* as facultative anaerobes. In the blood stream in which schistosomes reside, the absence of free O_*2*_ could drive these microbes towards schistosome microenvironments such as the tegument or the gastrodermis. In *S*. *japonicum* we also identified elevated levels of *Proteobacteria* on the parasite tegument, relative to the blood of an infected host (*p-value* = 0.044). *Bacteroidetes* and *Firmicutes* were also shown present in our study. While similar levels were evident in host blood, both with and without a schistosome infection, *Bacteroidetes* and *Firmicutes* were also associated with the parasite surface and gastrodermis, although at reduced levels compared with the surrounding blood. The potential benefits of these and other taxa require further evaluation. Our FISH labelling provided evidence for an association between bacteria and adult schistosome epithelial tissues. Notably, we found EUB labelling was detected both in the tegument and gastrodermis of female worms, whereas it was only observed in the gastrodermis of males, suggesting females may utilize host blood and associated microbiome differently to meet the needs of egg production. The observed labelling patterns were distinct to other reports using similar *in situ* hybridization methods to study schistosome-specific transcripts in sectioned adult worms [39, 40]. Expanding FISH to include more specific species or genera would be a significant first step to further confirm their presence within the parasite.

Reviewed by Abruzzi and Fried [41], animal and human studies have shown altered levels of bacteria, including *Mycobacteria*, *Helicobacter* and *Staphylococcus*, in host tissues such as the lungs and liver during schistosome infection. In our study, only *Staphylococcus* was found present in most samples, including the parasite tegument, although the levels were not statistically different compared with the surrounding blood, even when compared with blood samples from uninfected mice.

### Why do helminths alter their microbiomes?

Helminths immunomodulate the host innate and adaptive immune systems for their benefit [42]. Bacteria also utilise multiple strategies to modulate host immune responses [43], but it is unknown if any interactions between bacteria and schistosomes provide advantages to the parasite at the host-parasite interface. In an active schistosome infection, parasite eggs perforate the host intestinal wall leading to bacterial leakage [44], but the direct impact this has on the host blood microbiome has not yet been explored to any great extent.

Natural co-infections of schistosomes and *Salmonella* led in the 1980’s to *in vitro* experiments demonstrating bacterial attachment to the *S*. *mansoni* surface tegument [45]. The significance of these interactions was never fully revealed, although a more recent study indicated that *S*. *typhimurium* infection in mice reduces the number of adult schistosome worms [46]. One potential benefit for bacteria associating with the schistosome tegument has been explored by Barnhill and colleagues [47]. They acknowledged the commonality of concurrent infections between schistosomes and *Salmonella* commonly occurring in humans, which they expanded on using *in vitro* assays. Using isolated adult *S*. *mansoni* they demonstrated that bacteria attached to the parasite surface were protected from antibiotic therapy. An interaction between the bacteria and the schistosome appeared specific, since the presence of mammalian cells or a free-living flatworm, did not provide protection from the antibiotics. The benefit that a specific bacterial population may provide to the parasite has, however, not been considered. The use of modified *in vitro* culture conditions could be key to addressing this question.

Of the few taxa elevated in the parasite carcass, one was *Clostridium aminophilum* (*p-value* = 0.0079) normally associated with ruminants. *Clostridium* is a major pathogen of humans, which can produce toxins, but can, as an obligate fermenter, produce compounds that may be of benefit to the parasite. In the mammalian gut, microbiota are known to produce short chain fatty acids that are essential for the maintenance of epithelial cells [48], particularly in apical membrane fidelity [49]. The importance of membrane maintenance in schistosomes is central to tegumental and gastrodermal functions [13, 50]. Studies examining the urinary metabolomic features during experimental schistosomiasis mansoni found indicators of microbial impact via increased levels of *p*-cresol glucuronide which has been reported to be produced by species of *Clostridium* [51, 52]. This component is also produced by species of *Lactobacillus* which we detected in all four tissues. In an earlier study [52] a *S*. *mansoni*-infected mouse demonstrated that urine metabolites including *p*-cresol glucuronide were elevated. The authors associated these altered metabolites with an altered host microbiome. Considering our new findings, these schistosome-associated urine metabolites, including *p*-cresol, may result from elevated levels of *Clostridium* and *Lactobacillus* directly associated with the adult schistosome worms residing in the host blood. The potential of *p*-cresol in the blood by parasite-associated tegument bacteria, presenting in the urine, is supported by studies of rats where iv injections of these metabolites were excreted via the glomerulus [53]. Increased levels of *Lactobacillus* have been associated with other helminth models including *Heligmosomoides polygyrus* in the ileum and duodenum of mice [54, 55]. We present in this study novel sites of residence of *Lactobacillus* on the schistosome tegument and in the parasite gut.

Another notable carcass-associated taxon was *Prevotella* (*p-value* = 0.0054). *Prevotella copri* has been reported in the blood of a single clinical case of heart failure [56]. *Prevotella* is considered a commensal but a recent finding links it to host inflammatory responses [57]. This type of response from the host driven by the bacteria would be detrimental to the parasite but given that this taxon was associated with the carcass or gastrodermis, rather than the exposed tegument, could make this less detrimental to the parasite. *Prevotella copri* produces succinate, and has been shown to improve glucose homeostasis in humans in a mechanism thought to involve microbe-host interaction and communication [58]. One possible mechanism explored in mice suggested succinate acts as a glucose precursor which stimulates gluconeogenesis in the mammalian gut [59]. In addition to potential metabolic effects of *Prevotella-*produced succinate within the schistosome gut, studies of gut protozoa indicate an interaction between the pathosymbiotes producing succinate and host immunomodulation [60]. The authors suggested similar mechanisms could be produced in succinate-producing bacteria, and the possibility of schistosomes harbouring *Prevotella* could be a novel aspect associated with parasitism. Any potential advantage, either metabolic or immunological, provided as a result of *Prevotella* being present in the schistosome gut could be determined as an important future research avenue.

### Altered blood and bile microbiomes, and the impact of helminth infection

The presence of bacteria in the blood outside full sepsis is a contentious topic [61]. However, more information is arising challenging the previously held dogma that blood is generally sterile in a healthy individual [61, 62]. Many studies have reported the existence of circulating bacterial DNA but did not provide evidence for the presence of viable organisms. More recently, molecular techniques, in conjunction with classical aerobic and anaerobic microbial culture experiments, were able to characterise the human circulating microbiome [61, 62]. The impact of blood feeding GIT will be clearer with the breach in intestinal fidelity potentially leading to changes in the blood microbiome [63]. As discussed above, the breach of intestinal fidelity by schistosomes will be driven by the eggs of the parasite. The presence of specific bacteria in the blood as a result of an active schistosome infection is a new observation. The significance and functionality of the observed increase or decrease of blood taxa will, however, require considerable investigation. Future studies in mice and humans should explore the potential of comorbidities of schistosomiasis and the presence of elevated levels of bacteria in host blood.

Some bacterial species on the schistosome parasite surface could represent an additional immune-evasion mechanism. *Streptococcus*, *Haemophilus influenzae*, *Escherichia coli* K1, and *Neisseria meningitidi* all possess structures which prevent antibody adherence and complement insertion on their surfaces [64]. Of these taxa, *Escherichia coli* (not K1) is present on the tegument and carcass of the parasite (*p-value* = 4.5E-16). The secretions by Gram-negative bacteria may be another defence mechanism that could benefit schistosomes. The presence of the multi-laminated apical tegument membrane of adult schistosomes [13, 65, 66] could make the parasites resistant to bacterial toxins, but may perforate host cells, particularly those associated with the immune response.

*Schistosoma japonicum* and *S*. *mansoni* both cause hepato-intestinal fibrotic schistosomiasis, although direct impact on the host gallbladder or bile duct by eggs is rare [21]. Schistosomal cholecystitis is recognized as one of the rare gastrointestinal presentations of schistosomiasis. However, it is not clear whether the schistosome eggs trapped in the gallbladder walls may directly induce this type of acute inflammation and alter the bile microbiome [19]. Species that do have a more documented negative impact on the host gallbladder include *Fasciola hepatica*, *Opisthorchis viverrini*, *Clonorchis sinensis* and *Opisthorchis felineus*, all of which reside in the biliary system [67].

Bile has been analysed for the presence of microbial communities in animals [68, 69] and humans [17]. Most studies report increases in bacterial levels associated with gall bladder disease. The presence of bacteria within bile has also been reported in humans without any underlying co-pathology [17]. These include the bacterial Phyla Actinobacteria, Bacteroidetes, Firmicutes and Proteobacteria. We found all four of these Phyla present in bile samples although none were statistically altered as a result of schistosome infection.

The bile (also known as gall) content of patients infected with *O*. *felineus* were profiled for changes in the microbiome [70]. Changes observed in infected patients included the presence of *Cellulosimicrobium*, *Microlunatus*, *Mycoplana* and *Phycicoccus*, and elevated levels of *Bacteroides*, *Klebsiella*, *Leptotrichia*, *Lactobacillus*, *Rothia*, *Selenomonas* and *Treponema*. While none of these were observed in our study, it is notable that many of the taxa we did find were similarly increased in infected animals compared with controls, as well as some that were only evident during infection. Elevated levels of *Limnohabitans* (*p-value* = 0.031), *Fluviicola* (*p-value* = 0.022), *Clostridium* (*p-value* = 0.026) and *Curvibacter* (*p-value* = 0.013) genera were associated with bile taken from infected mice. These observations further strengthen the importance of the bile microbiome in helminth-infected mammals.

## Future directions and conclusions

Despite the new data presented here, key questions still need to be clarified that additional time course experiments of longer duration would help address. By sampling adult worms of *S*. *japonicum* before and after egg laying, an approach used in another schistosome microbiome study focusing on the host faecal environment [7, 71], the importance of the host intestine may be determined. The significance of the production of eggs, and the translocation across the intestinal lumen is potentially critical for the introduction of new bacteria into host blood and to the adult parasite. The application of single sex male only infections, where no eggs are produced, will also help to clarify this issue, and would help indicate which bacterial changes result from the host intestine becoming compromised [44] and what impact parasite pairing (male and female parasite infections) has on the parasite microbiome. Similarly, examining the incoming infectious cercarial stage may provide a further life cycle approach to understanding schistosome, bacteria and host interactions. In addition, it would be interesting to investigate the difference in both blood and intestinal microbiota in the infected animals after being treated with praziquantel or antibiotics. That may shed lights on the mechanism of drug action.

Our results highlight an underappreciated aspect of schistosome biology. The adult parasite, resident in the blood system of the definitive mammalian host, is active both in modulating the host immune system, and in accessing essential nutrients. An additional feature may be that the adult schistosome alters its interactions with the microbiome present in the blood. All three of these key phenomena may interact to ensure effective parasitism. We demonstrate that the microbiomes on the epithelial surfaces of adult schistosomes are distinct to that present in the host blood microenvironment where they reside. The increased concentration of some bacteria, as well as the absence of other bacterial taxa, on schistosome epithelial surfaces, represents a newly described microbial niche. We also consider the differences between the schistosome gastrodermis and tegument microbiomes provide another strong indicator that differences in functionalities exist between the two. As is frequently the case for a new area of research, many questions remain due to the complex interaction between the schistosome, the host and the microbial populations present. We anticipate that further exploration of this important area will provide new pointers for developing anti-schistosomiasis interventions exploiting the interactions between bacteria and schistosomes.

## Supporting information

S1 File Calypso v3 format csv (comma-separated values) file before decontam kitome normalisation. Abundance is presented. Column B = OTU assignments. Row 1, Column C-AZ = Sample ID (see S1 Table for corresponding sample descriptions. Corresponds to S3 File which is in biome format.
(CSV)

S2 File Calypso v3 format csv (comma-separated values) file after decontam kitome normalisation. Abundance is presented. Column B = OTU assignments. Row 1, Column C-AZ = Sample ID (see S1 Table for corresponding sample descriptions. Corresponds to S4 File which is in biome format.
(CSV)

S3 File Biome format file before decontam kitome normalisation. (BIOM)

S4 File Biome format file after decontam kitome normalisation. (BIOM)

S1 Table Metadata file containing- Sample ID, Label, Individual, Tissue and Infection, Tissue-Type and description. (CSV)

S2 Table ANOVA of host blood and bile samples, with both infected and uninfected host controls. Separate tabs contain Species, Genus and Phylum level data. Results include *p- value* based in Tissue and Infection status, False Discover Rate (FDR), adjusted *p- value* based in Tissue and Infection status, Paired *p- value* (Tukey), and mean abundance for each Tissue and Infection condition.
(XLSX)

S3 Table ANOVA of host bile samples only, both infected and uninfected controls. Separate tabs contain Species and Genus level data. Results include *p- value* based in Tissue and Infection status, False Discover Rate (FDR), adjusted *p- value* based in Tissue and Infection status, Paired *p- value* (Tukey), and mean abundance for each Tissue and Infection condition.
(XLSX)

S4 Table ANOVA of host blood in both infected and uninfected controls, and parasite samples tegument and carcass. Separate tabs contain Species, Genus and Phylum level data. Results include *p- value* based in Tissue and Infection status, False Discover Rate (FDR), adjusted *p- value* based in Tissue and Infection status, Paired *p- value* (Tukey), and mean abundance for each Tissue and Infection condition.
(XLSX)

S5 Table ANOVA of parasite samples tegument and carcass only. Separate tabs contain Species, Genus and Phylum level data. Results include *p- value* based in Tissue and Infection status, False Discover Rate (FDR), adjusted *p- value* based in Tissue and Infection status, Paired *p- value* (Tukey), and mean abundance for each Tissue source.
(XLSX)

S1 Fig Kitome normalisation of the data, presented as a non-clustered bar-chart. Major OTUs present in individual samples before (Top) and after (Bottom) decontam processing of data.
(TIF)

S2 Fig Major species, genus, family and phylum present in parasite tissue samples and fluids from infected and uninfected hosts. Host (left) and parasite (right) present variances until the phylum level, where differences become less prominent.
(TIF)

S3 Fig Examples of statistically significant bacterial species with elevated levels either in the host blood, or in the parasite tegument and/or carcass. (TIF)

## Abbreviations

CCA: Canonical correspondence analysis
FISH: fluorescent *in situ* hybridisation
GIT: gastro-intestinal trematodes
I: infected
M: Mouse
OTU: Operational taxonomic unit
PCoA: Principal Coordinates Analysis
RDA: Redundancy analysis
TBS: Tris-buffered saline
U: uninfected
USP: Universal Stress Protein domains
