# Supplementary figures and images for: Adult schistosomes have an epithelial bacterial population distinct from the surrounding mammalian host blood
Source: PLoS One. 2022 Jan 27;17(1):e0263188. doi: 10.1371/journal.pone.0263188 (PMC8794206; doi:10.1371/journal.pone.0263188)

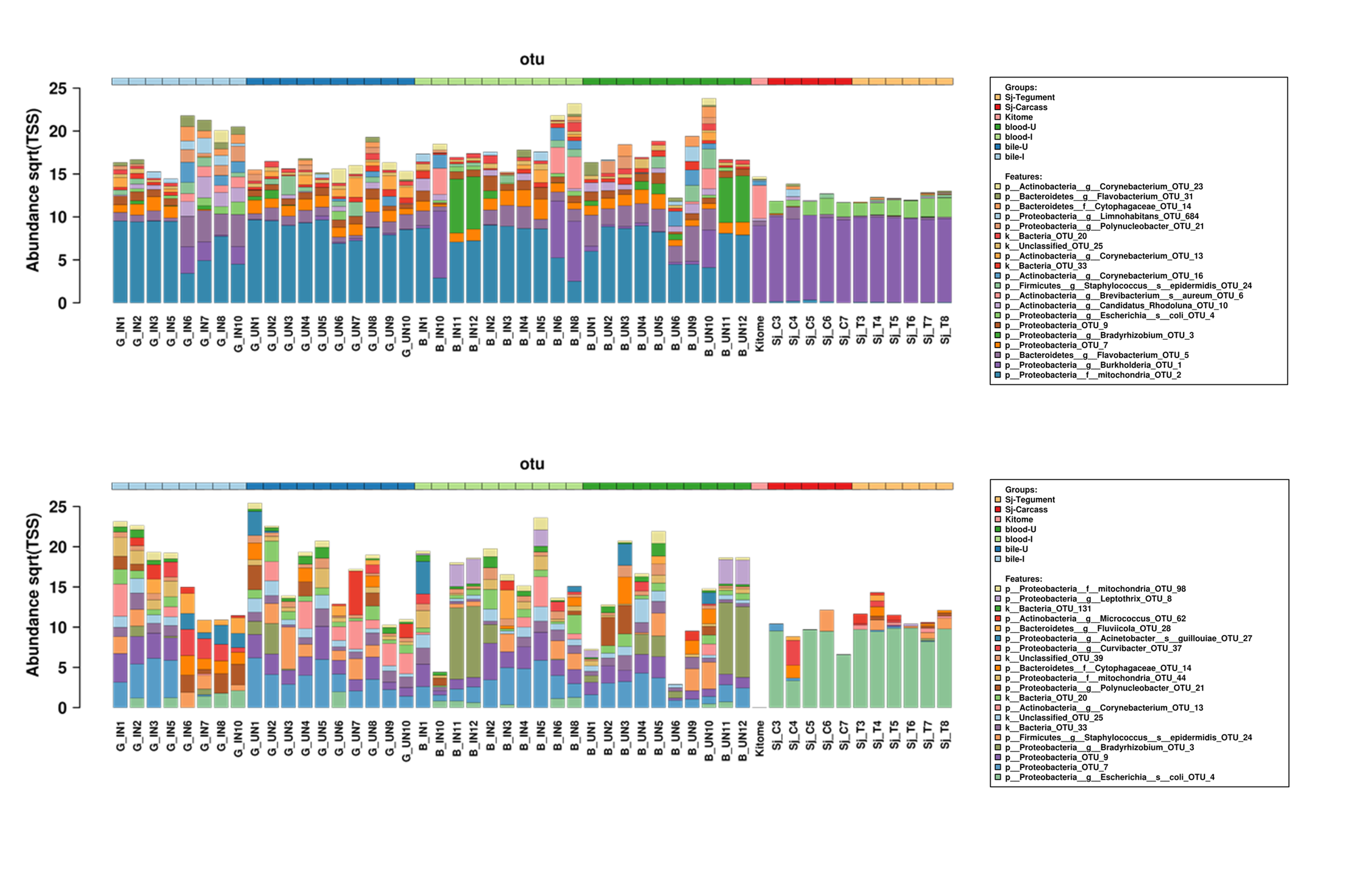

Supplement: S1 Fig — Major OTUs present in individual samples before (Top) and after (Bottom) decontam processing of data. (TIF) [file pone.0263188.s010.tif]

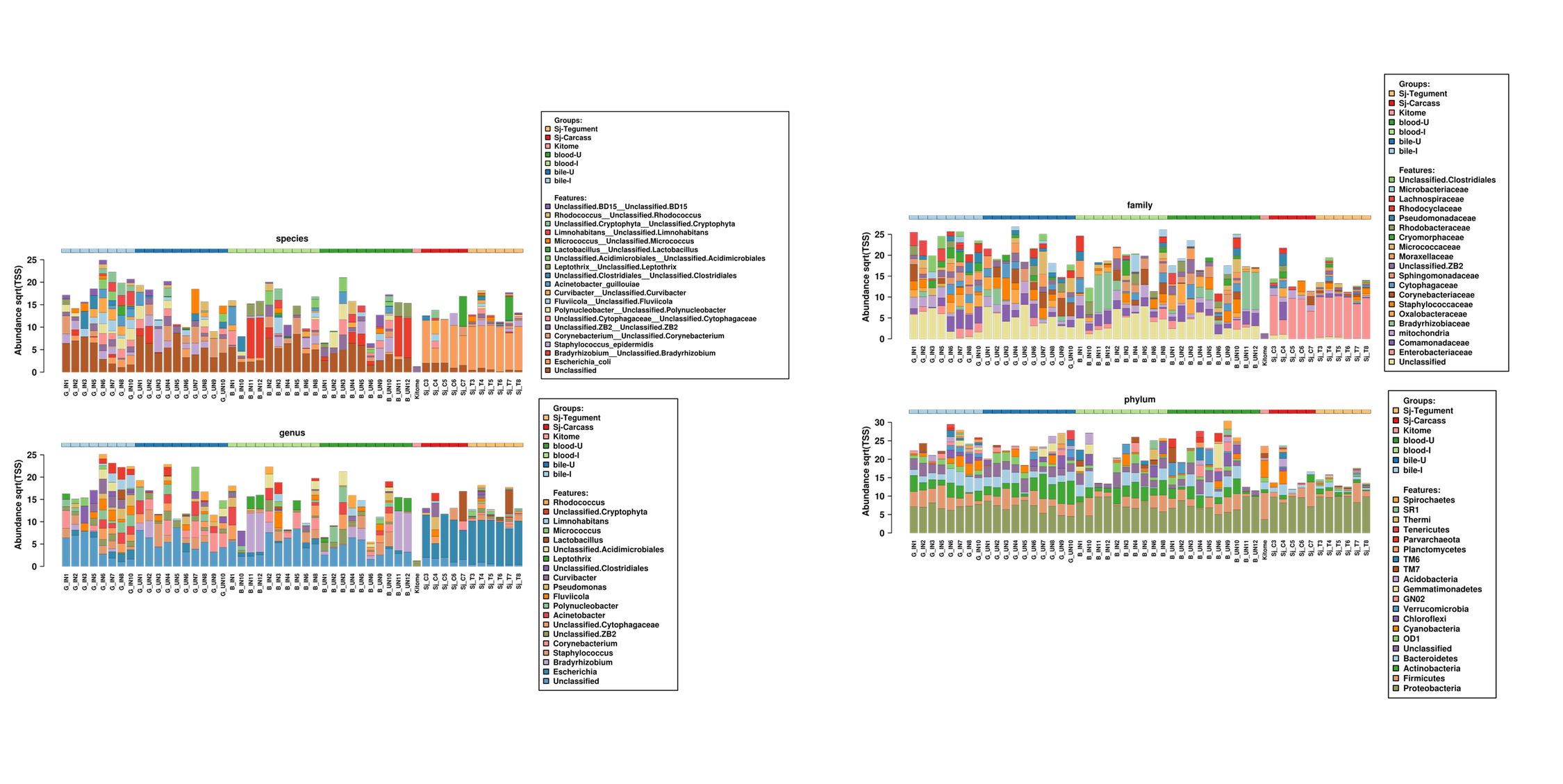

Supplement: S2 Fig — Host (left) and parasite (right) present variances until the phylum level, where differences become less prominent. (TIF) [file pone.0263188.s011.tif]

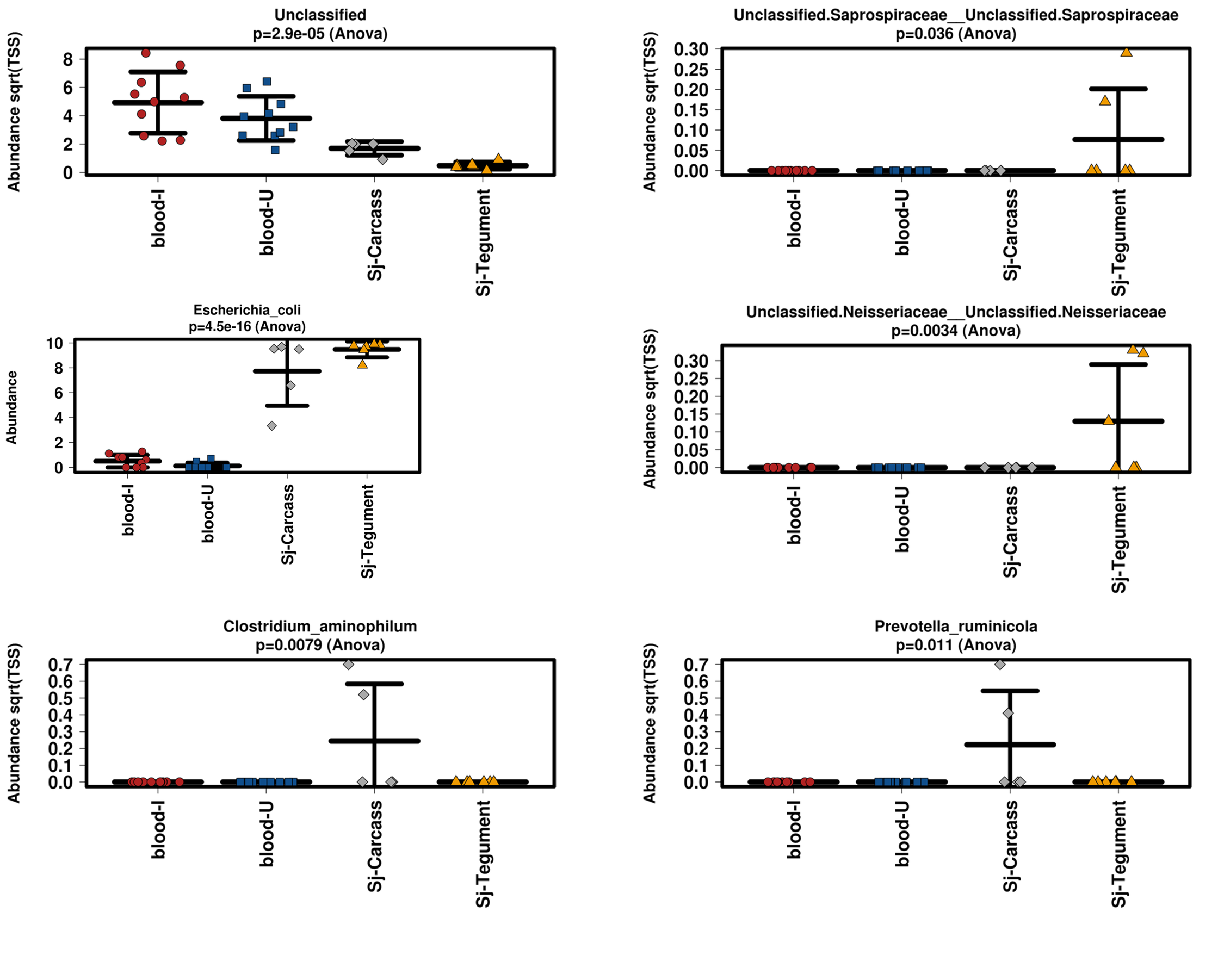

Supplement: S3 Fig — (TIF) [file pone.0263188.s012.tif]
